# Altered Preoperative Nutritional Status in Colorectal Cancer: A Not So Infrequent Issue

**DOI:** 10.1155/2020/5049194

**Published:** 2020-11-07

**Authors:** Javier Páramo-Zunzunegui, Araceli Ramos-Carrasco, Marcos Alonso-García, Rosa Cuberes-Montserrat, Gil Rodríguez-Caravaca, Manuel Durán-Poveda

**Affiliations:** ^1^Department of General Surgery and Digestive Diseases, Hospital Universitario de Móstoles, Madrid, Spain; ^2^International Doctoral Program, University Rey Juan Carlos, Madrid, Spain; ^3^Department of Endocrinology and Nutrition, Hospital Universitario de Móstoles, Madrid, Spain; ^4^Preventive Medicine Unit, Hospital Universitario Fundación Alcorcón, Alcorcón, Spain; ^5^Department of Preventive Medicine and Public Health, Health Sciences Faculty, Rey Juan Carlos University, Madrid, Spain; ^6^Department of General Surgery and Digestive Diseases, Hospital Universitario Rey Juan Carlos, Madrid, Spain

## Abstract

**Introduction:**

Malnutrition and weight loss in cancer patients is a common problem that affects the prognosis of the disease. In the case of CRC, malnutrition rates range between 30 and 60%.

**Objectives:**

Description of the preoperative nutritional status of patients diagnosed with colorectal neoplasia who will undergo surgery.

**Materials and Methods:**

A prospective observational study is performed.

**Results:**

Of 234 patients studied, we observed that 139 (59%) had some degree of nutritional risk. Of all of them, 44.9% (*N* = 47) had 1-2 points according to MUST and 25% (*N* = 27) had more than 2 points. No differences were found when studying nutritional risk according to the location of the neoplasm. It was observed that 2.15% of the patients were underweight, 51% overweight, and 23% obese. 19.4% of patients lost less than 5 kg in the 3–6 months prior to diagnosis, 20.7% lost between 5 and 10 kg, and 2.1% lost more than 10 kg. In asymptomatic patients, the weight loss was lower than in symptomatic patients, loss <5 kg, 8.2% vs. 22.8%, and loss 5–10 kg, 16.2% vs. 29.3%, with a value of *p* = 0.016. 5% (*N* = 7) of the patients had hypoalbuminemia record. 16.5% (*N* = 23) had some degree of prealbumin deficiency and 20.9% (*N* = 29) of hypoproteinemia. Symptomatic patients had more frequent analytical alterations, 1-2 altered parameters in 48.8% (*N* = 20) of asymptomatic vs. 61.2% (*N* = 22) in the symptomatic, *p* = 0.049.

## 1. Introduction

Colorectal cancer has an approximate incidence in Spain of 33 cases per 100,000 inhabitants/year [1], and is the third most common cancer, after breast and prostate cancer. Men are slightly more likely to develop colorectal cancer [2]. The most important risk factor is age, with 90% of cases diagnosed in patients over 50 [3]. The EUROCARE-5 study places the average 5-year survival of colon cancer at 57.1% and that of rectal cancer at 56.4% in Spain [4]. In Spain, and in line with established guidelines, population screening of the CRC is recommended with the faecal occult blood test (FOBT) every two years for people aged 50 to 74 [5]. In recent decades, the nutritional risk of patients undergoing colon or rectal surgery has been linked to the development of complications [6]. In the case of CRC, malnutrition rates range from 30 to 60% [7, 8]. Several nutritional assessment tests have been developed to detect and attempt to treat this clinical condition, such as the Malnutrition Screening Tool and Nutritional Risk Index (MUST) and the Patient-Generated Subjective Global Assessment (PG-SGA). Despite its extensive acceptance by the scientific community and proven evidence, its application in daily clinical practice is not without some degree of subjectivity and difficulty. The objective of this study is to establish the nutritional status of patients diagnosed with colon or rectal cancer who underwent surgery in 2017 and 2018.

## 2. Materials and Methods

Prospective observational study of 234 patients diagnosed with colon or rectal cancer surgically treated at the General and Digestive Surgery Department at the Móstoles University Hospital randomly selected and fulfilling preestablished inclusion criteria. Patients of both sexes who were over 18 years of age, undiagnosed with anorexia and bulimia, and willing to participate, excluding those with impossibility or refusal to respond to subjective questions, were selected. Nutritional assessment included anthropometric study (weight, size, and BMI), weight loss in the last 3–6 months, assessment with MUST nutritional screening tool, and analytical study of nutritional parameters (albumin, total proteins, prealbumin, retinol-binding protein (RBP), transferrin, and cholesterol). The criteria for patients with some degree of nutritional risk are detailed in (Table 1).

## 3. Results

All patients agreed to participate in the study. The 234 patients who came to their first appointment at the General and Digestive Department with a diagnosis of colorectal cancer were analysed. Of the 234 patients, we observed that 139 (59%) had some degree of nutritional risk. The average age of this group of patients was 69.9 years (DE = 9.5), with an interquartile range of 11.5. The sex ratios in the study were 65.5% men (*N* = 91) and 34.5% women (*N* = 48). The most common patient cormorbidities were high blood pressure (HBP) (53.3%), dyslipidemia (DL) (38.7%), and diabetes mellitus type I (17%). A total of 12.6% of patients had heart disease (heart failure, atrial fibrillation, or coronary heart disease) and 13.3% had chronic obstructive pulmonary disease (COPD) (Table 2). The data related to neoplasm location of the studied population are shown in (Table 2). The mean BMI of the patients was 27.33 (DE = 3.88). Stratifying by BMI groups, we observed that 3 patients (2.15%) were in underweight, 32 patients (23%) had normal weight, 72 (51.8%) were overweight, and 32 patients (23%) were obese. For the study of the weight loss variable, the following groups were defined: loss <5 kg, loss of between 5–10 kg, loss of ≥10 kg, and no weight loss. We thus observed that 48 patients (34.5%) had no weight loss, 27 (19.4%) lost less than 5 kg, 35 (20.7%) lost between 5 and 10 kg, 3 patients (2.1%) lost more than 10 kg, and 26 (18.7%) were unable to provide this information. Frequency analysis of symptomatic status of patients at diagnosis is shown in Table 2. The results observed when analysing the distribution of unintentional weight loss among patients according to their symptomatic status at the diagnosis (asymptomatic patients who were diagnosed via screening campaign vs. symptomatic patients) are shown in Table 3: loss of <5 kg, loss of 5–10 kg, and loss of ≥10 kg were more frequently observed in the group of symptomatic patients (22.4% (*N* = 22), 29.6% (*N* = 29), and 4% (*N* = 4), respectively). Statistically significant differences were found between the two groups with *p* = 0.016.

The mean and median of the nutritional biomarkers studied are shown in Table 3. We highlight that the 5% of patients (*N* = 7) had hypoalbuminemia (<3.5 g/dl), 16.5% (*N* = 23) had some degree of prealbumin deficiency (<21 mg/dL), 20.9% (*N* = 29) had hypoproteinemia (<6.4 g/dl), and RBP deficiency was detected in 2.9% (*N* = 4) of patients.

Statistically significant differences are found (*p* = 0.049) in the frequency distribution of the total number of analytical parameters altered according to the patient's symptomatic condition. In the asymptomatic (screening) group, 48.8% (*N* = 20) had 1 or 2 altered parameters. In the group of symptomatic patients, 63.3% (*N* = 62) had 1 or 2 altered analytical parameters and 24.5% (*N* = 24) had >2 parameters. Nutritional risk distribution according to the MUST score in patients with colorectal cancer is shown in Table 3.

## 4. Discussion

Malnutrition and weight loss in the cancer patient is a frequent problem that affects the prognosis of the disease [9]. The cancer patient presents a state of generalized hypermetabolism that, together with the systemic effects of the surgical aggression, results in an increased risk of complications [10]. Cancer-related inflammation, related to cytokines, small inflammatory proteins, and immune cells, is considered by clinicians as a sustained and perhaps inappropriate systemic reaction to malignancy that results in fevers, sweats, weight loss (the so-called B symptoms), and a range of other paraneoplastic symptoms [11].

Malnutrition in the surgical patient is associated with higher rates of postsurgical complications, mortality, prolongation of hospital stay, and, therefore, health costs [12]. These conclusions have been the result of numerous articles that were published since the second half of the 20th century. Butterworth was the pioneer in 1974 in stimulating the scientific community in the promotion of cost-effective strategies for the identification and recognition of malnutrition in the hospital patient [13]. It was in the 80s when Buzby et al. and Harvey et al. created prognostic indexes to assess the effects of malnutrition in the hospitalized patient [14, 15]. Coinciding with this period, the American Society for Parenteral and Enteral Nutrition (ASPEN) was founded in 1975 and, later, the European Society for Clinical Nutrition and Metabolism (ESPEN) in 1980.

According to ESPEN, tumors of the gastrointestinal tract, head and neck, and liver and lung are the ones that are most associated with a higher nutritional risk [16]. In this study, we have observed an increase nutritional risk in 139 patients of a total of 234 colorectal cancer patients (59%), taking into account the inclusion criteria described in Table 1, during the years 2017 and 2018. The scientific literature provides malnutrition rates of between 40 and 80% in cancer patients [17]. The work carried out by Segura et al. (NUPAC study) determines different degrees of prevalence of malnutrition according to the location of the tumor. Thus, esophageal and stomach tumors are those with the highest prevalence with 57.7% and 50%, respectively [18, 19]. In the case of CCR, rates range between 30 and 60%. The BMI of the study population was 27.33, with no statistical differences between the two cohorts of the study (symptomatic vs. asymptomatic patients). We have observed 2.15% of patients with BMI <18.5, 51.8% in the overweight group, and 23% in the obesity group. These rates are discretely higher than those offered by the International Agency for Research on Cancer (IARC): 15.8% obese and 33.7% overweight in the general population. This may be due to the fact that our population is a nonrepresentative sample of the general population. On the one hand, we study patients with an established diagnosis of colorectal neoplasia, and, on the other, the average age of the sample is greater than the population's. However, in “The heavy burden of obesity” prevalence study, rates of 47.5% of overweight and 28.5% of obesity were observed in general population of Spain [20].

Among all the patients, we observed 27 patients (19.4%) unintentionally lost less than 5 kg, 35 (20.7%) lost between 5 and 10 kg and 3 (2.1%) lost more than 10 kg. In the classic scientific literature, we find that the proportion of patients who present some degree of weight loss at diagnosis ranges between 15 and 40% [21]. Burden et al found that 77% of patients diagnosed with CRC have some degree of weight loss and in 20% of these patients the weight loss exceeds 10% [22].

We have found statistically significant differences in the distribution of weight loss according to symptomatic status. Patients who were diagnosed in the screening campaign, and who were asymptomatic, had lower weight loss rates than the symptomatic patients. In other words, 81.5% of patients who unintentionally lost <5 kg and 77.1% who lost between 5 and 10 kilos belonged to the symptomatic group.

There are some studies in the Pubmed® database that research the impact of weight loss in the prognosis of CRC and other malignancies [23]. We have not found studies which specifically relate weight loss rates to the symptomatic state of the disease. This may be due to the fact that there are few CRC screening programs with the trajectory of our country's, Prevecolon® CRC Screening Campaign in Madrid.

According to nutritional biomarkers, we have identified that 5% of the patients had hypoalbuminemia. A retrospective, multicenter observational study detected an incidence of hypoalbuminemia of 17% in CRC [24]. A prospective observational study related albumin levels with the development of complications after cardiac surgery, observing after the multivariate study that hypoalbuminemia, was the risk factor most strongly associated with morbidity and mortality after surgery with an OR 0.68, 95% CI 0.56–0.60, *p* = 0.001 [25]. More specifically, in colorectal surgery, hypoalbuminemia is also related to the development of postoperative complications [26]. Hennessey et al., in a multicenter study, identified hypoalbuminemia it as an independent risk factor for the development of SSI [27].

The rest of the nutritional biomarkers currently have a controversial utility, although some review had identified albumin, prealbumin, hemoglobin, total cholesterol, and total protein, as useful biomarkers for adult malnutrition assessment of a surgical patient [28]. We observed some degree of prealbumin deficit in 10% (*N* = 23) and hypoproteinemia in 12.4% (*N* = 29) of all CRC patients. Statistically significant differences (*p* = 0.049) are found after association of the total number of altered analytical parameters and symptomatic status at diagnosis. In the group of symptomatic patients, 26.5% (*N* = 62) of the patients had 1 or 2 altered analytical parameters, and 8% (*N* = 18) had >2 parameters. Various nutritional assessment tools associate the deficit of nutritional biomarkers and malnutrition [29]. According to the nutritional data observed in our study and literature, a protocolized and extended nutritional assessment should be performed in CRC patients due to the high incidence of alterations and their association to surgery complications.

## 5. Conclusions

Malnutrition has a substantial clinical and socioeconomic significance. It impacts on morbidity, mortality, length of hospital stay, and costs. There is an extended perception and concern about the importance of the nutritional status assessment in gastrointestinal neoplasms. However, CRC is still an underestimated pathology in this area, and more implication of clinicians should be performed.

## Figures and Tables

**Table 1:** Inclusion criteria in nutritional risk.

*Age (years)*	≥70
and/or	

*MUST*	≥1 (intermediate or high)
and/or	

*Degree of biochemical alteration*	≥2 altered parameters
and/or	

*Surgical complexity*	Left hemicolectomy and rectum resection

**Table 2:** Demographic characteristics of the population at nutritional risk.

*Sex*	*N* (%)
Female	45 (34.6)
Male	85 (65.4)

*Age (SD)*	69.9 (SD = 9.5)

*Comorbidities*	

HTA	72 (55.3)
Diabetes mellitus	33 (25.4)
Dyslipidemia	52 (4 0)
Heart disease	17 (13)
EPOC	18 (13.8)
Thyroid disease	14 (10.8)

*Toxic habits*	
Active smoker	16 (12.3)
Former smoker	65 (50)
Alcohol occasional	26 (86.7)
Alcohol moderate	32 (14.6)
Alcohol abuse	2 (1.5)

*ASA*	
ASA I	5 (3.8)
ASA II	78 (60)
ASA III	36 (27.7)
ASA IV	2 (1.5)

*Neoplasm location*	
Right colon	51 (36.7)
Transverse colon	3 (2.2)
Left colon	47 (33.8)
Sigma	28 (20.1)
Rectum	10 (7.2)

*Symptomatic status at diagnosis*	
Nonsymtomatic (FOB+)^*∗*^	40 (28.8)
Rectorrhagia	43 (31)
Anemia	29 (20.9)
Constitutional syndrome	5 (3.6)
Constitutional syndrome and anemia	14 (10)
Others	8 (5.7)

*BMI (SD)*	27.33 (SD = 3.88)

*MUST score*	

0 points	31 (30)

1-2 points	47 (44.9)

>2 points	27 (25)

^*∗*^(FOB) faecal occult blood positive in screening campaign prevecolon.

**Table 3:** Analysis of nutritional variables by group of patients according to symptomatic state at diagnosis.

	Asymptomatic (screening)	Symptomatic	*p* value
*BMI*	27.3 (SD = 3)	27.4 (SD = 4)	0.9

*Weight loss*	*N* (%)	*N* (%)	*p* value
No weight loss	18 (27.7)	26 (40.6)	0.016
Weight loss ≤5 kg	9 (13.8)	15 (23.4)	
Weight loss 5–10 kg	12 (18.5)	21 (32.8)	
Weight loss ≥10 kg	1 (1.5)	2 (3.1)	
Not asked	25 (38.5)	0 (0)	

*Biochemical parameter study*	Media (SD)	Media (SD)	*p* value
Albumin	4.5 (0.7)	4.1 (0.5)	0.004
Prealbumin	25.5 (5.8)	23.9 (5.7)	0.3
Serum total protein	6.8 (0.6)	6.7 (0.8)	0.6
RBP	4.4 (1)	4.2 (0.9)	0.5
Lymphocytes	2.8 (2.7)	2.4 (1.9)	0.4
Cholesterol	185 (34)	161 (38)	0.002

*Total number of analytical parameters altered*	Asymptomatic *N* (%)	Symptomatic *N* (%)	*p* value
No altered parameter	13 (46.4)	15 (53.6)	0.049
1-2 altered parameters	20 (25)	60 (75)	
≥2 altered parameters	4 (18.2)	18 (81.8)	

## Data Availability

The data can be available from the corresponding author upon request.

## Abbreviations

IARC: International Agency for Research on Cancer
ASPEN: American Society for Parenteral and Enteral Nutrition
CRC: Colorectal cancer
DL: Deciliter
ESPEN: European Society for Clinical Nutrition and Metabolism
BMI: Body mass index
MUST: Malnutrition screening tool and nutritional risk index
RBP: Retinol bound protein
PG-SGA: Patient-generated subjective global assessment
FOBi: Fecal occult blood.
